# Clinical application of intraoperative somatic tissue oxygen saturation for detecting postoperative early kidney dysfunction patients undergoing living donor liver transplantation: A propensity score matching analysis

**DOI:** 10.1371/journal.pone.0262847

**Published:** 2022-01-21

**Authors:** Jaesik Park, Sangmin Jung, Sanghoon Na, Ho Joong Choi, Jung-Woo Shim, Hyung Mook Lee, Sang Hyun Hong, Min Suk Chae

**Affiliations:** 1 Department of Anesthesiology and Pain Medicine, Seoul St. Mary’s Hospital, College of Medicine, The Catholic University of Korea, Seoul, Republic of Korea; 2 Department of Anesthesiology and Pain Medicine, Incheon St. Mary’s Hospital, College of Medicine, The Catholic University of Korea, Seoul, Republic of Korea; 3 Department of Surgery, Seoul St. Mary’s Hospital, College of Medicine, The Catholic University of Korea, Seoul, Republic of Korea; Imperial College Healthcare NHS Trust, UNITED KINGDOM

## Abstract

**Background:**

Somatic tissue oxygen saturation (SstO_2_) is associated with systemic hypoperfusion. Kidney dysfunction may lead to increased mortality and morbidity in patients who undergo living donor liver transplantation (LDLT). We investigated the clinical utility of SstO_2_ during LDLT for identifying postoperative kidney dysfunction.

**Patients and methods:**

Data from 304 adults undergoing elective LDLT between January 2015 and February 2020 at Seoul St. Mary’s Hospital were retrospectively collected. Thirty-six patients were excluded based on the exclusion criteria. In total, 268 adults were analyzed, and 200 patients were 1:1 propensity score (PS)-matched.

**Results:**

Patients with early kidney dysfunction had significantly lower intraoperative SstO_2_ values than those with normal kidney function. Low SstO_2_ (< 66%) 1 h after graft reperfusion was more highly predictive of early kidney dysfunction than the values measured in other intraoperative phases. A decline in the SstO_2_ was also related to kidney dysfunction.

**Conclusions:**

Kidney dysfunction after LDLT is associated with patient morbidity and mortality. Our results may assist in the detection of early kidney dysfunction by providing a basis for analyzing SstO_2_ in patients undergoing LDLT. A low SstO_2_ (< 66%)_,_ particularly 1 h after graft reperfusion, was significantly associated with early kidney dysfunction after surgery. SstO_2_ monitoring may facilitate the identification of early kidney dysfunction and enable early management of patients.

## Introduction

Living donor liver transplantation (LDLT) is a critical treatment for patients with end-stage liver disease (ESLD). Kidney dysfunction is one of the most common complications after liver transplantation (LT), affecting short- and/or long-term outcomes. Therefore, it is essential to identify intraoperative risk factors for the development of kidney dysfunction [1]. Many factors affect the development of kidney dysfunction after LT, such as the model for end-stage liver disease (MELD) score, age, sex, body mass index (BMI), chronic kidney disease, and diabetes mellitus (DM) [2]. Because of intraoperative hemodynamic fluctuations, renal tissue may be susceptible to hypoperfusion that subsequently leads to kidney functional impairment. Therefore, continuous monitoring of organ/tissue perfusion and saturation is valuable to avoid functional and structural injury [3].

Cerebral oximetry is a technique developed for detecting regional cerebral oxygen saturation using near-infrared (NIR) spectroscopy. NIR light contacts the hemoglobin beneath the sensor, which causes the light spectrum to change. This light returns to the detector of the oximeter and regional hemoglobin oxygen saturation (rSO_2_) [cerebral (SctO_2_) and somatic (SstO_2_) tissue oxygen saturations] can be determined [4]. Cerebral oximetry has been applied during major surgeries, such as cardiac surgery and carotid endarterectomy [5]. A lower rSO_2_ in patients undergoing cardiac surgery is associated with a higher risk of morbidities (such as necrotizing enterocolitis), high lactate levels, and low mixed venous oxygen saturation. These findings suggest that a lower rSO_2_ may be a clinical marker for poor systemic perfusion and saturation [6, 7]. In a previous report of adults undergoing cardiac surgery, the SstO_2_ value recorded from the thenar muscle of the hand was associated with the development of acute kidney injury (AKI). In that report, the SstO_2_ 20 min after cardiopulmonary bypass had a cut-off value of ≤ 54.5% and acceptable area under the receiver operating characteristic curve (AUC) values of around 70% [8].

In patients undergoing LT, SstO_2_ monitoring may play a role in detecting early complications, such as graft rejection or abdominal compartment syndrome [9–11]. Multi-site applied NIR spectroscopic monitoring, including SctO_2_ and SstO_2_, is available to facilitate the detection of systemic/peripheral hypoperfusion [12, 13]. In a previous LT study [14], low arterial oxygen content was significantly associated with postoperative kidney dysfunction. Because low SstO_2_ is closely related to inadequate oxygen delivery [15], continuous and noninvasive SstO_2_ monitoring may be useful for assessing the risk of early kidney dysfunction during surgery.

The role of SstO_2_ in kidney function monitoring during LDLT surgery has still not been established; thus, we investigated the clinical utility of intraoperative SstO_2_ for identifying kidney dysfunction during the early postoperative period.

## Patients and methods

### Ethical considerations

The present study was approved by the Institutional Review Board and Ethics Committee of Seoul St. Mary’s Hospital (approval number: KC20RISI0176), Seoul, Republic of Korea on April 6, 2020, and the study was performed according to the principles of the Declaration of Helsinki. The requirement for informed consent was waived because of the retrospective nature of the study.

### Study population

Data from 304 adults (aged > 19 years) undergoing elective LDLT between January 2015 and February 2020 at Seoul St. Mary’s Hospital were retrospectively collected from the electronic medical records system. Exclusion criteria included a preoperative history of kidney dysfunction (e.g., dialysis, chronic kidney disease [estimated glomerular filtration rate (eGFR) < 60 mL/min/1.73 m^2^], or hepatorenal syndrome), and missing laboratory data. Thirty-six patients were excluded based on the exclusion criteria, of which twelve (33.3%) were excluded because of missing data. In total, 268 adults were analyzed, and 200 were matched via 1:1 propensity score (PS) matching (S1 Fig).

### Oximetry monitoring

All patients were monitored using the INVOS 5100c oximeter (Medtronic, Minneapolis, MN, USA) which consists of a light source and detectors. Based on the difference in NIR light absorption between oxygenated and unoxygenated hemoglobin, the oximeter system calculates hemoglobin saturation of the blood, in the brain or other tissues beneath the sensor, using the Beer-Lambert law [4, 16].

In the present study, a somatic sensor was placed on the forearm on the opposite side of the peripheral venous line (S2 Fig). The cerebral sensor was placed laterally on the skin of the patients’ forehead on the same side as the somatic sensor. Somatic (SstO_2_) and cerebral (SctO_2_) oximetry values were recorded five times after inducing anesthesia (immediately after anesthetic induction [T0], immediately after liver dissection [T1], at the time of inferior vena cava [IVC] partial clamping [T2], 5 min after graft reperfusion [T3], and 1 h after graft reperfusion [T4]).

### Definition of early kidney dysfunction

Kidney function was evaluated based on the eGFR, calculated using the Modification of Diet in Renal Disease formula: eGFR = 175 × standardized serum creatinine^-1.154^ × age^-0.203^ × 1.212 (if black) × 0.742 (if female) [17]. The baseline eGFR was estimated the day before surgery, and serial eGFRs were measured on postoperative days (PODs) 1–7. Based on the eGFR [18], kidney function was classified as normal (eGFR ≥ 90 mL/min/1.73 m^2^), mild dysfunction (eGFR 89–60 mL/min/1.73 m^2^), or moderate dysfunction (eGFR 59–30 mL/min/1.73 m^2^). In our study, early kidney dysfunction was defined as the lowest eGFR < 60 mL/min/1.73 m^2^ during the first week after surgery.

### Living donor liver transplantation

LT and general anesthesia were performed by expert surgeons and anesthesiologists, respectively. The surgical procedure and anesthetic management were described in detail in our previous study [19]. The surgical technique was identical for the entire study population [20]. The piggyback surgical technique was performed using the right liver lobe and the middle hepatic vein tributaries (segments 5 and 8) were connected to the middle hepatic vein of the recipient using prosthetic grafts. Vascular anastomoses (hepatic vein, portal vein, and hepatic artery) and bile duct reconstruction were performed. Cross-clamping of the inferior vena cava (IVC) was performed for the hepatic and portal vein reconstruction and hepatic vascular flow (portal venous flow and hepatic arterial resistive index) were evaluated with Doppler ultrasonography (Prosound SSD-5000; Hitachi Aloka Medical, Tokyo, Japan). Splenectomy, splenic artery ligation was performed as required.

Balanced anesthesia was applied with proper hemodynamic management (mean arterial pressure ≥ 65 mm Hg and central venous pressure ≤ 10 mm Hg) under hemodynamic monitoring. According to the transfusion guidelines, [21] packed red blood cells were transfused to reach a hematocrit ≥ 25%, and coagulation factors (fresh frozen plasma, single-donor platelets, and cryoprecipitate) were transfused based on laboratory findings or thromboelastography.

As calcineurin inhibitor was used as an immunosuppressant after surgery. The trough calcineurin inhibitor level was maintained between 7 and 10ng/mL for the first postopearative month. Other immunosuppressants (mycophenolate mofetil and prednisolone), which were administered according to our hospital’s LDLT protocol, were gradually adjusted and tapered after LDLT [20, 22].

### Perioperative recipient and donor findings

The preoperative recipient findings included age, sex, BMI, comorbidities (hypertension and DM), ejection fraction, eGFR, MELD score, hepatic decompensation parameters (encephalopathy [West-Haven grade I–II] [23], ascites, and varix), and laboratory data (hematocrit, white blood cell [WBC] count, and percentages of neutrophils and lymphocytes). The intraoperative recipient findings included operation time, requirement for a norepinephrine infusion (≥ 0.05 μg/kg/min), averages of the hemodynamic parameters (systolic and diastolic blood pressure, heart rate, and central venous pressure), blood product requirements (packed red blood cells, fresh frozen plasma, single donor platelets, and cryoprecipitate transfusions), laboratory data (hemoglobin and lactate), hourly fluid infusion, and hourly urine output. The postoperative findings in recipients included the eGFR and kidney dysfunction (eGFR < 60 mL/min/1.73 m^2^). Donor findings included age, sex, BMI, graft-recipient weight ratio, total ischemic time, and donor graft fatty change.

### Statistical analyses

We compared the levels of tissue oxygen saturation (SstO_2_ and SctO_2_) between the normal and kidney dysfunction groups using analysis of covariance (ANCOVA). Oxygen saturation at each stage was also compared using the Mann-Whitney *U* test and *χ*^*2*^ or Fisher’s exact test, as appropriate. The predictive accuracy of the models was evaluated based on the AUC. The optimal cut-off of the SstO_2_ for the prediction of early kidney dysfunction was determined using the AUC. In addition, 1:1 PS matching was used to correct any imbalance in confounders between the low and high SstO_2_ groups. After matching, we compared perioperative recipient and donor graft factors using the Mann-Whitney *U* test and *χ*^*2*^ or Fisher’s exact test, as appropriate. The association between low SstO_2_ 1 h after graft reperfusion (< 66%) and postoperative early kidney dysfunction was evaluated by multivariate logistic regression analyses after adjusting for the PS and intraoperative factors, and odds ratios (ORs) with 95% confidence intervals (CIs) were calculated. Continuous data are presented as medians and interquartile range (IQR), and categorical data are presented as frequencies and proportions. A *p*-value < 0.05 was considered significant in all analyses. Statistical analyses were performed with SPSS for Windows (ver. 24.0; IBM Corp., Armonk, NY, USA), R software (ver. 2.10.1; R Foundation for Statistical Computing, Vienna, Austria), and MedCalc for Windows (ver. 11.0; MedCalc, Ostend, Belgium).

## Results

### Comparison of oxygen saturation between patients with and without early kidney dysfunction

**Fig 1:**
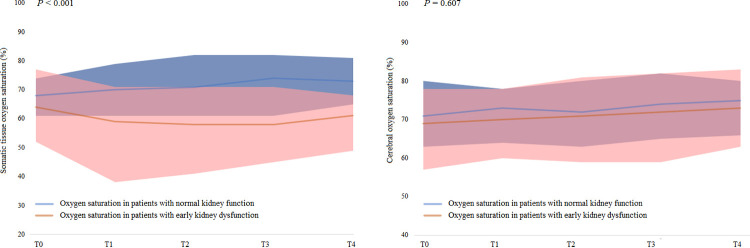
Comparison of oxygen saturation between patients with and without early kidney dysfunction at each stage. The curve and shaded area represent median and IQR values of oxygen saturation, respectively. T0 = immediately after anesthetic induction; T1 = immediately after liver dissection; T2 = IVC partial clamping; T3 = 5 min after graft reperfusion; T4 = 1 h after graft reperfusion.

Patients with early kidney dysfunction showed significantly lower intraoperative SstO_2_ than those without (ANCOVA, *p* < 0.001; Fig 1 and S1 Table). However, SctO_2_ did not differ between patients with and without kidney dysfunction (ANCOVA, *p* = 0.607).

### Association of the area under the receiver operating characteristic curve with intraoperative SstO_2_ and change in SstO_2_ reflecting postoperative early kidney dysfunction in all patients

**Fig 2:**
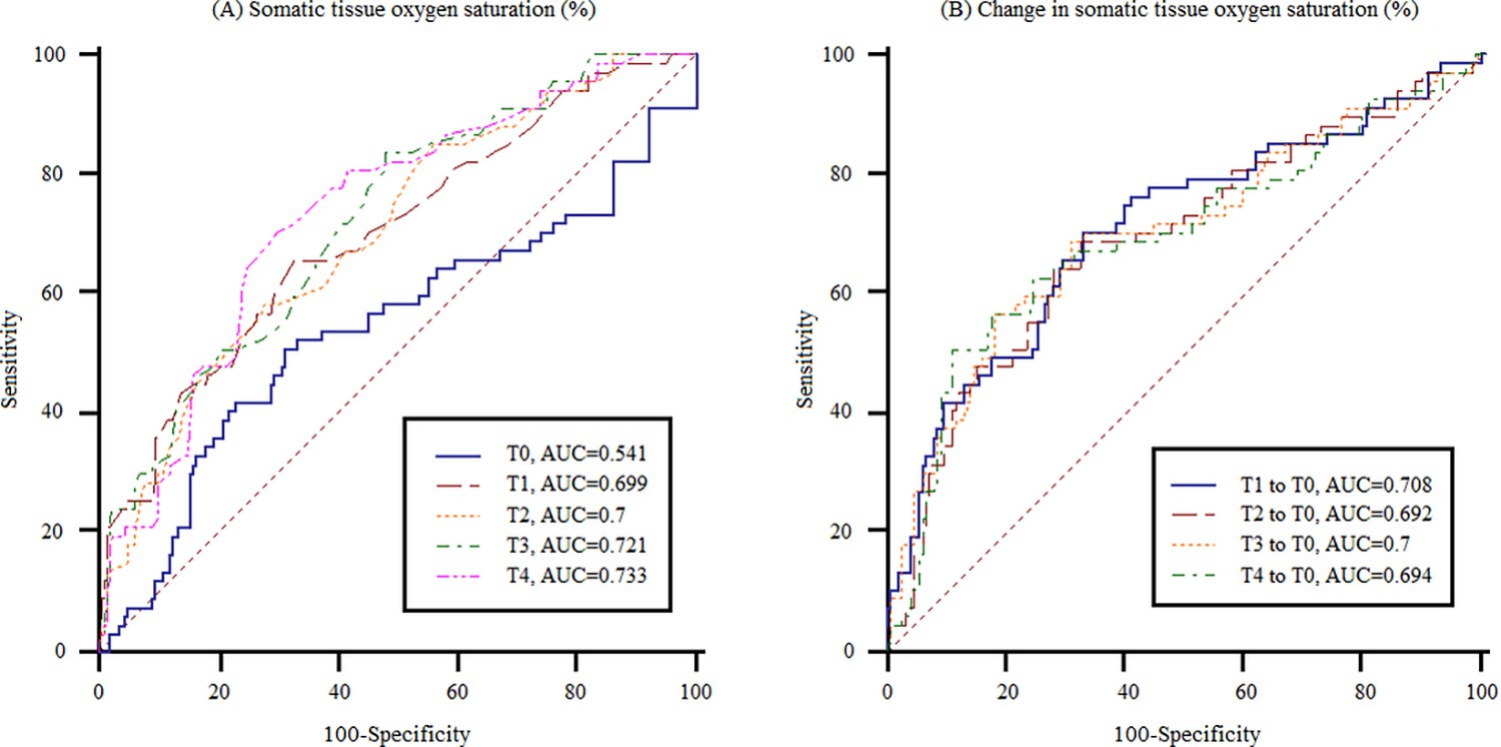
Comparison of the AUC values of SstO_2_ (A) and the AUC for the change in SstO_2_ compared to saturation at the time of anesthesia induction (B) for postoperative early kidney dysfunction, at each stage. T0 = immediately after anesthetic induction; T1 = immediately after liver dissection; T2 = IVC partial clamping; T3 = 5 min after graft reperfusion; T4 = 1 h after graft reperfusion.

Regarding the SstO_2_ AUC values at different times (Fig 2(A)), that at T4 (1 h after graft reperfusion) was most significantly associated with early kidney dysfunction. A low SstO_2_ (< 66%) at T4 was significantly associated with early kidney dysfunction (AUC: 0.733, 95% CI: 0.675–0.785, sensitivity: 70.1%, specificity: 70.1%, *p* < 0.001 in the predictive model). Regarding the receiver operating characteristics curves of the change in SstO_2_ compared to saturation at T0, the difference in oxygen saturation between T0 and T1 was significantly associated with early kidney dysfunction (AUC: 0.708, 95% CI: 0.65–0.762, sensitivity: 70.1%, specificity: 67.2%, *p* < 0.001 in the predictive model; Fig 2(B)).

### Comparison of pre-and intraoperative recipient and donor graft factors before and after PS matching

Significant differences were observed in the preoperative factors (MELD score, ascites, hematocrit, and WBC count) and donor-graft parameters (BMI, graft-to-recipient weight ratio, and total ischemic time; Table 1) between the high and low SstO_2_ groups. No significant differences were detected between the groups after PS matching.

**Table 1:** Preoperative recipient and donor-graft findings for the high and low somatic tissue oxygen saturation (1 h after graft reperfusion) groups, before and after PS-matching.

	Before PS-matching analysis				After PS-matching analysis			
Group	H-SstO_2_ (≥ 66%)	L-SstO_2_ (< 66%)	*p*-value	SD	H-SstO_2_ (≥ 66%)	L-SstO_2_ (< 66%)	*p*-value	SD
n	164	104			100	100		
***Preoperative recipient findings***								
Age (years)	55 (50–60)	55 (48–61)	0.975	0.047	54 (50–60)	55 (48–61)	0.819	0.091
Sex (female)	40 (24.4%)	31 (29.8%)	0.327	0.118	24 (24.0%)	30 (30.0%)	0.339	0.131
Body mass index (kg/m^2^)	24.2 (22.2–26.6)	24.0 (21.8–26.9)	0.659	–0.024	24.4 (22.1–27.2)	24.0 (21.8–27.0)	0.478	–0.077
Hypertension	41 (25.0%)	20 (19.2%)	0.272	–0.146	22 (22.0%)	20 (20.0%)	0.728	–0.051
Diabetes mellitus	41 (25.0%)	33 (31.7%)	0.23	0.144	30 (30.0%)	32 (32.0%)	0.760	0.043
Calcineurin inhibitor level	8.2 (7–9.3)	7.9 (6.6–9.3)	0.242	–0.035	7.9 (7.2–8.7)	7.9 (6.7–9.3)	0.726	–0.064
Ejection fraction (%)	64.1 (62.0–66.0)	64.4 (62.0–66.0)	0.709	–0.028	64.0 (62.0–66.0)	64.4 (62.0–66.0)	0.770	–0.062
eGFR (mL/min/1.73m^2^)	88.3 (71.8–106.5)	84.0 (64.8–112.1)	0.564	–0.035	90.1 (70.0–106.5)	84.0 (66.5–112.1)	0.754	–0.063
MELD score (points)	9 (5–19)	17 (9–26)	<0.001	0.468	12.0 (7.5–22.4)	17.4 (8.8–26.3)	0.149	0.199
Encephalopathy (West-Haven criteria I or II)	73 (44.5%)	55 (52.9%)	0.181	0.167	48 (48.0%)	52 (52.0%)	0.572	0.080
Esophageal varix	45 (27.4%)	26 (25.0%)	0.659	–0.056	24 (24.0%)	26 (26.0%)	0.744	0.046
Ascites	67 (40.9%)	76 (73.1%)	<0.001	0.723	63 (63.0%)	72 (72.0%)	0.174	0.202
Hematocrit (%)	32.0 (26.6–37.3)	29.0 (24.5–34.2)	0.001	–0.445	29.8 (25.3–37.0)	29.0 (24.5–34.3)	0.131	–0.240
White blood cell count (× 10^9^/L)	4.4 (3.1–6.6)	5.7 (3.1–9.5)	0.017	0.276	4.9 (3.1–7.6)	5.7 (3.0–8.9)	0.293	0.122
Neutrophil (%)	59.4 (49.9–70.5)	64.3 (55.4–76.4)	0.006	0.131	62.6 (51.4–75.0)	64.0 (55.4–76.3)	0.217	0.190
Lymphocyte (%)	26.9 (15.2–34.7)	18.8 (10.2–29.5)	<0.001	–0.455	21.6 (12.0–32.2)	19.0 (10.2–30.0)	0.156	–0.210
***Donor-graft findings***								
Age (years)	35 (26–44)	35 (28–40)	0.789	0.006	35 (26–44)	35 (28–40)	0.939	–0.027
Sex (female)	50 (30.5%)	29 (27.9%)	0.649	–0.058	31 (31.0%)	29 (29.0%)	0.758	–0.044
Body mass index (kg/m^2^)	20.2 (18.2–21.6)	20.3 (20.2–23.2)	0.014	0.278	20.3 (19.0–23.2)	20.3 (20.0–23.2)	0.397	0.101
Graft-recipient-weight-ratio (%)	1.2 (1.0–1.5)	1.3 (1.1–1.8)	0.021	0.324	1.3 (1.0–1.6)	1.3 (1.1–1.8)	0.387	0.146
Total ischemic time (min)	76 (57–99)	89 (64–170)	0.001	0.383	78.5 (58–110)	86 (63–167)	0.048	0.215
Fatty change (%)	4.9 (1.0–5.0)	4.9 (1.0–5.0)	0.036	0.243	4.9 (1.0–5.0)	4.9 (1.0–5.0)	0.368	0.068

**Abbreviations:** PS, propensity score; H-SstO_2_, group with high somatic tissue oxygen saturation; L-SstO_2_, group with low somatic tissue oxygen saturation; eGFR, estimated glomerular filtration rate; MELD, model for end-stage liver disease.

**Note**: Values are expressed as median (interquartile range) or number (proportion).

### Correlation between low SstO_2_ 1 h after graft reperfusion and early postoperative kidney dysfunction in PS-matched patients

A low SstO_2_ (< 66%) 1 h after graft reperfusion was significantly associated with the development of early kidney dysfunction in PS-matched patients (Table 2). After adjustment for the PS and intraoperative factors, a low SstO_2_ remained an independent predictor of early kidney dysfunction (*p* = 0.001).

**Table 2:** Association between low somatic tissue oxygen saturation (< 66%) 1 h after graft reperfusion and postoperative early kidney dysfunction in PS-matched patients.

	*β*	Odds ratio	95% confidence interval	*p*-value
L-SstO_2_	1.234	3.437	1.802–6.555	<0.001
L-SstO_2_ adjusted for PS	0.966	2.627	1.319–5.230	0.006
L-SstO_2_ adjusted for PS and intraoperative factors^§§^	1.356	3.881	1.807–8.337	0.001

**Abbreviations**: L-SstO2, low somatic tissue oxygen saturation; PS, propensity score

^§§^Intraoperative factors included the operation time, requirement for norepinephrine infusion (≥ 0.05 μg/kg/min), average systolic and diastolic blood pressure, heart rate, central venous pressure, requirement for packed red blood cells, fresh frozen plasma, single donor platelet and cryoprecipitate transfusions, average hemoglobin and lactate levels, hourly fluid infusion and hourly urine output.

### Comparison of the incidence of postoperative early kidney dysfunction between PS-matched patients with high and low SstO_2_ 1 h after graft reperfusion

Significantly more patients in the low SstO_2_ group (< 66%) suffered early kidney dysfunction (eGFR < 60 mL/min/1.73 m^2^) than in the high SstO_2_ group (≥ 66%) (*p* < 0.001; Table 3).

**Table 3:** Comparison of the incidence of postoperative early kidney dysfunction between PS-matched patients with high and low somatic tissue oxygen saturation 1 h after graft reperfusion.

Group	H-SstO_2_ (≥ 66%) (n = 100)	L-SstO_2_ (< 66%) (n = 100)	*p-*value
Normal kidney function	82 (82.0%)	57 (57.0%)	<0.001
Early kidney dysfunction	18 (18.0%)	43 (43.0%)	

**Abbreviations**: PS, propensity score; H-SstO2, group with high somatic tissue oxygen saturation; L-SstO2, group with low somatic tissue oxygen saturation.

**Note**: Values are expressed as number and proportion (%).

### Comparison of the proportions of kidney dysfunction between PS-matched patients with high and low SstO_2_ 1 h after graft reperfusion

Significantly more patients suffered kidney dysfunction (eGFR < 60 mL/min/1.73 m^2^) during the first week in the low SstO_2_ group (< 66%) after surgery than in the high SstO_2_ group (≥ 66%) (Table 4).

**Table 4:** Comparison of the proportions of kidney dysfunction between PS-matched patients with high and low somatic tissue oxygen saturation 1 h after graft reperfusion.

Group	H–SstO_2_ (≥ 66%) (n = 100)	L–SstO_2_ (< 66%) (n = 100)	*p*
**Kidney dysfunction (eGFR < 60 mL/min/1.73m** ^**2**^ **)**			
Preoperative day	0 (0.0%)	0 (0.0%)	–
Postoperative day			
POD 1	15 (15.0%)	35 (35.0%)	0.001
POD 2	11 (11.0%)	33 (33.0%)	<0.001
POD 3	11 (11.0%)	31 (31.0%)	0.001
POD 4	10 (10.0%)	26 (26.0%)	0.004
POD 5	9 (9.0%)	19 (19.0%)	0.051
POD 6	7 (7.0%)	17 (17.0%)	0.03
POD 7	6 (6.0%)	15 (15.0%)	0.048

**Abbreviations**: PS, propensity score; H-SstO2, group with high somatic tissue oxygen saturation; L-SstO2, group with low somatic tissue oxygen saturation.

**Note**: Values are expressed as number and proportion (%).

### Correlation between intraoperative somatic tissue oxygen saturation (%) and hourly urine output during liver transplantation

The somatic tissue oxygen saturation (%) at times T1–T4 was significantly associated with the hourly urine output (all *p* < 0.001, S2 Table).

## Discussion

The main finding of this study was that intraoperative SstO_2_ may have clinical utility to identify patients at risk of a decrease in eGFR < 60 mL/min/1.73 m^2^ during the first week after LDLT. A low SstO_2_ (< 66%) 1 h after graft reperfusion had higher predictive accuracy for early kidney dysfunction than values measured in other intraoperative phases. The low SstO_2_ (< 66%) in PS-matched patients, adjusted for PS and intraoperative factors, was approximately four-fold more strongly associated with early postoperative kidney dysfunction. A decline in the SstO_2_ compared to that measured immediately after inducing anesthesia was also related to kidney dysfunction. Thus, SstO_2_ itself, and deterioration therein, could also be important for predicting kidney dysfunction.

Intraoperative SstO_2_ monitoring has been widely performed in pediatric patients, and is usually measured in the renal, splanchnic, or thoracic beds because of the anatomical features (i.e., thin skin and subcutaneous adipose tissue). Pediatric cohort studies suggest that SstO_2_ is closely associated with systemic oxygenation, transfusion of red blood cells, and circulatory indices. Patients with a lower SstO_2_ level require more extracorporeal membrane oxygenation, inspired nitrogen, and mechanical ventilation than those with a higher SstO_2_ level [13, 24–26]. Pediatric renal SstO_2_ is an emerging and reliable non-invasive tool to identify early loss of kidney function. In cardiac surgery with cardiopulmonary bypass and an oximeter sensor at the level of the kidney (T10–L2) for renal oximetry, infants (aged < 1 year) with prolonged lower renal SstO_2_ values (i.e., oximetry < 65% and a relative decrease therein > 25%) during surgery and the first 48 h postoperatively showed more frequent AKI than those with higher values. Additionally, renal SstO_2_ may be better correlated with AKI compared to conventional laboratory markers (i.e., serum creatinine, urea and cystatin C, and urinary neutrophil gelatinase-associated lipocalin) [27].

Few studies have monitored SstO_2_ in the trunk beds of adults, including the renal beds, compared to the pediatric setting. One cardiac study reported that the intraoperative duration of renal SstO_2_ < 55% was associated with the postoperative occurrence of AKI [28]. However, because of the thicker skin and subcutaneous fatty tissues on the body trunk of adults than children, the SstO_2_ sensors are typically placed over skeletal muscles (i.e., the thenar eminence, forearm, or calf area) [29]. In a previous study of adults undergoing cardiac surgery, the SstO_2_ recorded from the thenar muscle of the hand was associated with the development of AKI. In that report, the SstO_2_ 20 min after cardiopulmonary bypass had a cut-off value of ≤ 54.5% and acceptable AUC values of around 70% [8]. Intraoperative tissue oxygenation status may be a composite marker of the balance between oxygen supply and consumption, which is largely determined by hemodynamic perfusion, a venous-weighted mixture of venous and arterial hemoglobin-oxygen saturation, and anesthetic-suppressed or surgical stress-related elevation of the metabolic rate [30].

SstO_2_ is an emerging marker of early postoperative complications in patients undergoing LT; however, SstO_2_ measurement sites differ among LT studies [9–11]. In an intensive care unit study by Civantos *et al*., the oximetry sensor was placed on the dermatome overlying the liver allograft [9]. SstO_2_ was significantly associated with the cardiac index, hemoglobin, and APACHE II score. Shiba *et al*. reported that a reduction in SstO_2_ (monitored on the dermatome overlying the liver allograft) was associated with an increased risk of acute graft rejection [10]. Hu *et al*. reported that full drainage of ascites during LT led to an increased leg SstO_2_; however, IVC clamping and abdominal hypertension caused a significant reduction in the leg SstO_2_. Somatic leg desaturation was more strongly associated with postoperative complications than arm and cerebral desaturation [11]. In our study, the SstO_2_ was measured at the forearm, which is more easily adjusted than a trunk bed. A lower SstO_2_ (measured at the forearm 1 h after graft reperfusion) increased the likelihood of early postoperative kidney dysfunction. SstO_2_ measured at the forearm provides an estimate of hemoglobin oxygen saturation in the mixed arterial, capillary, and venous blood in the tissue bed being probed, and is largely determined by the balance between organ/peripheral tissue oxygen consumption/supply and perfusion [31]. LT patients require large resuscitation and inotrope infusion volumes for vascular homeostasis; thus, SstO_2_ measured at the forearm may represent organ/peripheral tissue microcirculation, which is closely related to cardiac and systemic resistive indices [32, 33]. Large vital sign fluctuations have been observed within 1 h after liver graft reperfusion, and vasopressors and volume loading are frequently used to restore hemodynamics. Peripheral vascular tone declines (which may contribute to systemic hypotension) because of a huge surge in the circulation of inflammatory mediators from the liver graft, which may lead to organ/tissue hypoperfusion and desaturation [34]. Therefore, the lowest SstO_2_ level measured at the forearm 1 h after graft reperfusion may be a clinical marker of the lowest level of renal perfusion.

Some limitations of this study should be discussed. First, because cerebral oximetry was developed for detecting hypoperfusion of the brain, there is no established reference value for detecting hypoperfusion of other organs. Although a low SstO_2_ has been associated with organ dysfunction in previous studies [9, 27, 33], further study is required to validate the level of SstO_2_ for other organs, particularly the kidneys. Second, the somatic oximeter sensor was placed on the forearm in this study, and not on the renal parenchymal surface. Therefore, although SstO_2_ may reflect tissue oxygen perfusion status, it does not directly reflect the renal perfusion or saturation conditions. Therefore, further studies are required to determine the predictive value of SstO_2_ according to monitoring site. Third, there are important differences between liver transplants from living and deceased donors. In previous studies, LT with grafts from deceased donors were more than twice as strongly associated with postoperative AKI [35]. Further studies are required to validate the predictive value of SstO_2_ for kidney dysfunction in LT from deceased donors.

## Conclusion

Our results should increase the accuracy of detection of early kidney dysfunction by providing a basis for analyzing intraoperative SstO_2_ in patients undergoing LDLT. A low level of SstO_2_ during LDLT_,_ particularly 1 h after graft reperfusion, was significantly associated with early kidney dysfunction after surgery. When a low SstO_2_ is detected, meticulous monitoring and efforts to improve the possible causes of compromised tissue perfusion (hypovolemia, hypotension, or shock) are likely important. SstO_2_ monitoring provided additional information that may facilitate early management of LT patients who are vulnerable to kidney dysfunction.

## Supporting information

S1 Fig Study flow diagram. (TIF)

S2 Fig A near-infrared spectroscopy probe, positioned on the left or right forearm. (TIF)

S1 Table Comparison of changes in the (A) somatic tissue and (B) cerebral oxygen saturation levels between patients with and without early kidney dysfunction.
(DOC)

S2 Table Correlation between the intraoperative somatic tissue oxygen saturation (%) and hourly urine output during liver transplantation. (DOC)

S1 Raw data Data set on the association between low somatic tissue oxygen saturation (< 66%) 1 h after graft reperfusion and postoperative early kidney dysfunction in PS-matched patients. (XLSX)

## Abbreviations

LDLT: living donor liver transplantation
AKI: acute kidney injury
AUC: area under the receiver operating characteristic curve
BMI: body mass index
SctO_2_: cerebral tissue oxygen saturation
ESLD: end-stage liver disease
eGFR: estimated glomerular filtration rate
POD: postoperative day
MELD: the model for end-stage liver disease
IQR: interquartile range
IVC: inferior vena cava
LT: liver transplantation
NIR: near-infrared
PS: propensity score
rSO_2_: regional hemoglobin oxygen saturation
SstO_2_: somatic tissue oxygen saturation
OR: odds ratio
CI: confidence interval
WBC: white blood cell
