# Supplementary figures and images for: Clinical application of intraoperative somatic tissue oxygen saturation for detecting postoperative early kidney dysfunction patients undergoing living donor liver transplantation: A propensity score matching analysis
Source: PLoS One. 2022 Jan 21;17(1):e0262847. doi: 10.1371/journal.pone.0262847 (PMC8782411; doi:10.1371/journal.pone.0262847)

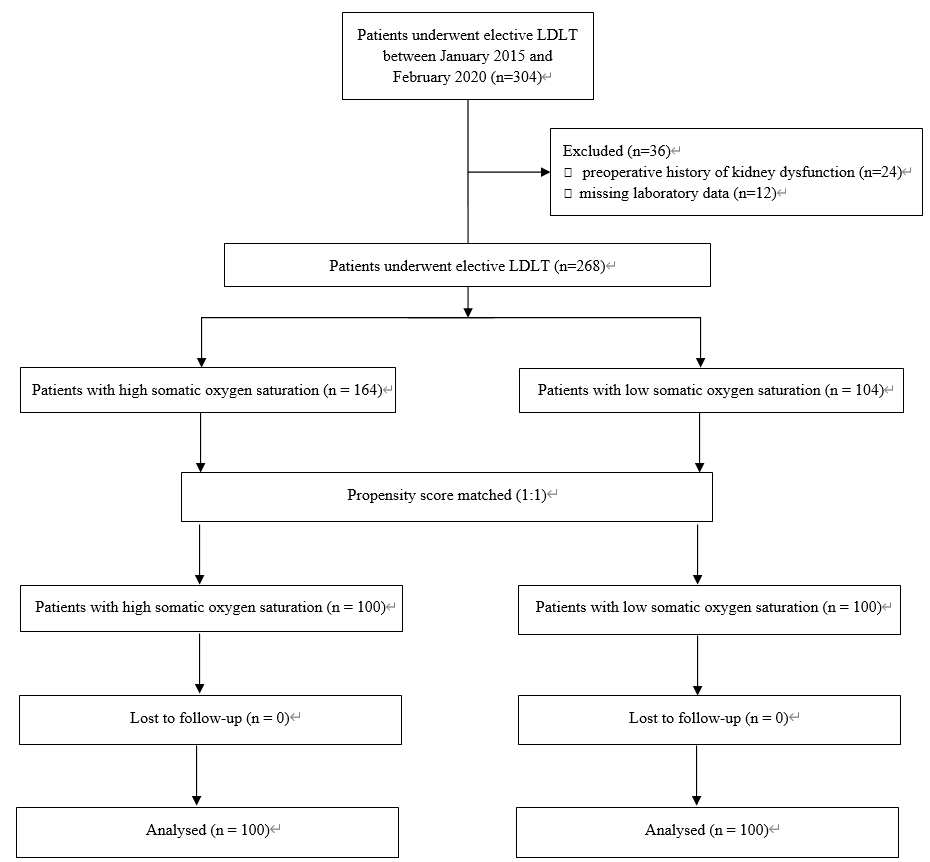

Supplement: S1 Fig — (TIF) [file pone.0262847.s001.tif]

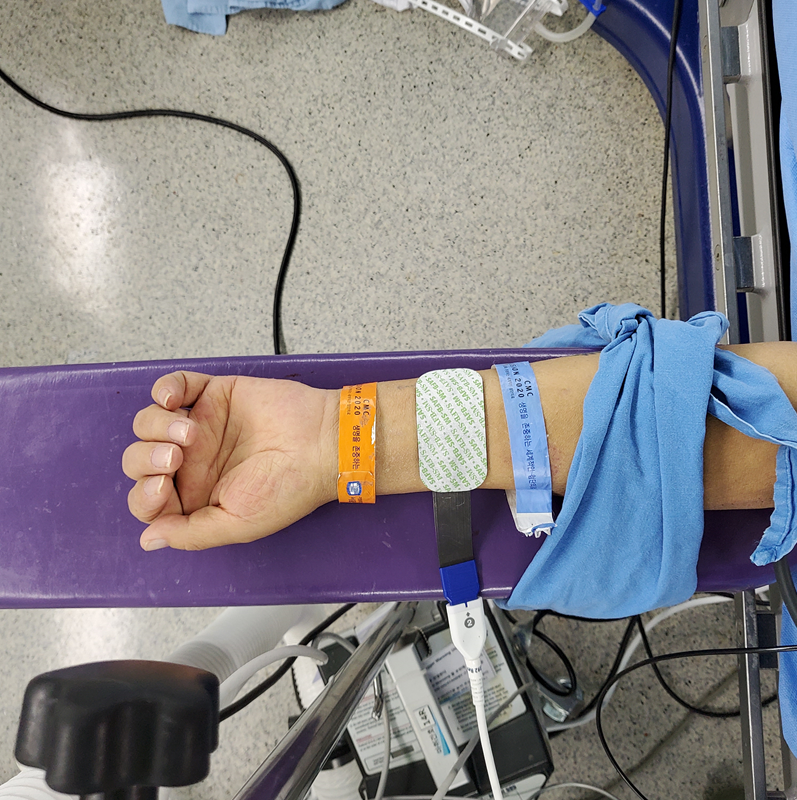

Supplement: S2 Fig — (TIF) [file pone.0262847.s002.tif]
