# Supplementary material for: Clinical application of intraoperative somatic tissue oxygen saturation for detecting postoperative early kidney dysfunction patients undergoing living donor liver transplantation: A propensity score matching analysis
Source: PLoS One. 2022 Jan 21;17(1):e0262847. doi: 10.1371/journal.pone.0262847 (PMC8782411; doi:10.1371/journal.pone.0262847)
Supplement: S1 Table — Comparison of changes in the (A) somatic tissue and (B) cerebral oxygen saturation levels between patients with and without early kidney dysfunction. (DOC) [file pone.0262847.s003.doc]

**S1 Table. Comparison of changes in the (A) somatic tissue and (B) cerebral oxygen saturation levels between patients with and without early kidney dysfunction.**

| **Group** | **Normal kidney function** | **Early kidney dysfunction** | ***p*** |
| --- | --- | --- | --- |
| **n** | 201 | 67 |  |
| **(A) Somatic tissue oxygen saturation (%)** |  |  |  |
| T0 | 68 (61 - 74) | 64 (52 - 77) | 0.311 |
| T1 | 70 (61 - 79)^†††^ | 59 (38 - 71)^†††^ | <0.001 |
| T2 | 71 (61 - 82)^†††^ | 58 (41 - 71)^†††^ | <0.001 |
| T3 | 74 (61 - 82)^†††^ | 58 (45 - 71)^††^ | <0.001 |
| T4 | 73 (65 - 81)^†††^ | 61 (49 - 68)^††^ | <0.001 |
| **(B) Cerebral oxygen saturation (%)** |  |  |  |
| T0 | 71 (63 - 80) | 69 (57 - 78) | 0.145 |
| T1 | 73 (64 - 78) | 70 (60 - 78) | 0.492 |
| T2 | 72 (63 - 80) | 71 (59 - 81) | 0.966 |
| T3 | 74 (65 - 82) | 72 (59 - 82)^†^ | 0.776 |
| T4 | 75 (66 - 80) | 73 (63 - 83)^†^ | 0.936 |

*p<0.05, **p≤0.01, ***p≤0.001 between two groups; †p<0.05, ††p≤0.01, †††p≤0.001 based on the level immediately after anesthetic induction.

T0 = immediately after anesthetic induction; T1 = immediately after liver dissection; T2 = IVC partial clamping; T3 = 5 min after graft reperfusion; T4 = 1h after graft reperfusion

**NOTE:** Values are expressed as median and interquartile.
