# Supplementary material for: Clinical application of intraoperative somatic tissue oxygen saturation for detecting postoperative early kidney dysfunction patients undergoing living donor liver transplantation: A propensity score matching analysis
Source: PLoS One. 2022 Jan 21;17(1):e0262847. doi: 10.1371/journal.pone.0262847 (PMC8782411; doi:10.1371/journal.pone.0262847)
Supplement: S2 Table — (DOC) [file pone.0262847.s004.doc]

**S2 Table. Correlation between the intraoperative somatic tissue oxygen saturation (%) and hourly urine output during liver transplantation.**

| **Group** | **Spearman’s Rho** | ***p*** |
| --- | --- | --- |
| T0 | -0.062 | 0.312 |
| T1 | -0.298 | <0.001 |
| T2 | -0.300 | <0.001 |
| T3 | -0.331 | <0.001 |
| T4 | -0.349 | <0.001 |

T0 = immediately after anesthetic induction; T1 = immediately after liver dissection; T2 = IVC partial clamping; T3 = 5 min after graft reperfusion; T4 = 1h after graft reperfusion
